# Clinical Characteristics and In-Hospital Outcomes of Traumatic Bladder Rupture: An 11-Year Retrospective Cohort Study at a Single Regional Trauma Center

**DOI:** 10.3390/jcm15187072

**Published:** 2026-09-11

**Authors:** Jaeik Jang, Myung Jin Jang, Kang Kook Choi, Soon Ki Min, Wu Seong Kang, Gil Jae Lee, Seung Hwan Lee, Jayun Cho, Byungchul Yu

**Affiliations:** 1 Department of Trauma Surgery, Gachon University Gil Medical Center, Incheon 21565, Republic of Korea; blanc7654@gmail.com (J.J.); wuseongkang@gilhospital.com (W.S.K.);; 2 Department of Traumatology, Gachon University College of Medicine, Incheon 21565, Republic of Korea

**Keywords:** urinary bladder, bladder rupture, trauma, pelvic fracture, treatment outcome

## Abstract

**Background/Objectives**: Traumatic bladder rupture often accompanies pelvic fracture, making it uncertain whether differences between extraperitoneal bladder rupture (EPBR) and intraperitoneal bladder rupture (IPBR) reflect the rupture site itself or overall trauma burden. Prior multi-institutional evidence has focused on EPBR. We therefore examined whether rupture site was associated with hospital length of stay after accounting for concomitant pelvic fracture and injury severity. **Methods**: We retrospectively reviewed 46 adults with definite traumatic bladder rupture treated at a single regional trauma center from January 2014 through December 2024. Rupture site was the primary exposure, hospital length of stay was the primary outcome, and intensive care unit (ICU) length of stay was the secondary outcome. Parsimonious exploratory log-linear models included rupture site, concomitant pelvic fracture, and Injury Severity Score (ISS). **Results**: Twenty-one patients had IPBR, 25 had EPBR, and 26 had concomitant pelvic fracture. In the adjusted primary-outcome analysis, rupture site was not clearly associated with hospital length of stay (EPBR versus IPBR adjusted ratio, 1.02; 95% CI, 0.59–1.76). The secondary adjusted analysis likewise showed no clear association with ICU length of stay (adjusted ratio for ICU days + 1, 1.31; 95% CI, 0.77–2.21). In unadjusted comparisons, pelvic fracture was observed more frequently with EPBR (72.0% versus 38.1%; *p* = 0.021; FDR q = 0.078), and EPBR was associated with longer hospital stay (median, 44.0 versus 24.0 days; *p* = 0.024; FDR q = 0.078) and ICU stay (8.0 versus 4.0 days; *p* = 0.007; FDR q = 0.037). Among 42 surgically treated patients, EPBR was associated with a longer admission-to-repair interval (adjusted ratio for days + 1, 2.21; 95% CI, 1.28–3.82). **Conclusions**: After adjustment for concomitant pelvic fracture and ISS, rupture site was not clearly associated with hospital or ICU length of stay. The conditional admission-to-repair finding may reflect complex trauma-care pathways rather than diagnostic delay. The frequent coexistence of bladder rupture and pelvic fracture reinforces the clinical importance of careful bladder assessment in patients with severe pelvic trauma.

## 1. Introduction

Traumatic bladder rupture is uncommon, although its reported frequency varies according to the study population and case definition. In a 10-year single-center cohort, definite bladder rupture occurred in 0.36% of adult blunt-trauma admissions [1], whereas broader estimates place bladder injury at approximately 1.6% of blunt abdominal trauma [2]. These estimates are not directly comparable because the broader category of bladder injury may include contusion or partial injury, whereas the present study was restricted to definite traumatic bladder rupture. It usually follows high-energy blunt trauma and is frequently accompanied by pelvic fracture or other major associated injuries [1,3,4]. Because early trauma care prioritizes immediately life-threatening head, chest, abdominal, and pelvic injuries, lower urinary tract injury can be missed or diagnosed late, especially in patients with pelvic fractures or polytrauma [3,5,6]. Delayed recognition may contribute to infection, persistent urinary extravasation, fistula formation, and prolonged recovery [4,6,7]. Current guidelines recommend dedicated retrograde cystography—either conventional or CT cystography—with active bladder filling when bladder injury is suspected; passive excretory-phase bladder filling is insufficient to exclude injury [8]. In a recent retrospective cohort of 698 patients with pelvic fracture, lower urinary tract injuries occurred in 7.7%; all affected patients had unstable pelvic fractures, and fracture pattern was associated with bladder-injury severity in men [9].

Traumatic bladder rupture is generally categorized as intraperitoneal bladder rupture (IPBR) or extraperitoneal bladder rupture (EPBR). This distinction is clinically important because it guides management. IPBR typically requires operative repair, whereas uncomplicated EPBR can often be managed nonoperatively with catheter drainage [4,10,11]. In contrast, complex EPBR, including bladder-neck involvement, associated rectal or vaginal injury, planned pelvic-fracture fixation, bony spicules, or persistent urinary extravasation, may require operative repair [2,12,13,14,15]. In this guideline context, persistent urinary extravasation refers to continued bladder contrast leakage documented on follow-up cystography after catheter drainage rather than leakage observed at the initial diagnosis. Catheter drainage for 2–3 weeks is standard for uncomplicated EPBR, and operative repair may be considered when healing has not occurred after more than 4 weeks [2].

Guideline recommendations provide a framework for management, but local practice is shaped by injury severity, pelvic-fracture management, timing of trauma operations, and available surgical expertise. Contemporary cohort studies illustrate this clinical heterogeneity. In the multi-institutional MiGUTS cohort of 157 patients with EPBR, 79% had a concomitant pelvic fracture and 43% underwent initial operative repair; common reasons included injury severity or bladder-neck involvement, identification during laparotomy, and concern regarding pelvic hardware contamination [12]. When EPBR coexists with a pelvic fracture requiring internal fixation, management remains individualized. Retrospective evidence suggests that operative bladder repair may reduce pelvic hardware infection in selected patients, whereas contemporary cohorts continue to show variable practice and mixed complication findings [12,14,15]. Recent single-center data likewise document substantial variation in diagnostic and management pathways for traumatic bladder injury [16].

MiGUTS provided important multi-institutional evidence regarding management patterns and complications among patients with EPBR but did not include patients with IPBR [12]. Consequently, whether differences in in-hospital outcomes between IPBR and EPBR reflect the anatomical rupture site itself or the burden of associated trauma remains uncertain. By including both rupture types over an 11-year period and evaluating rupture site together with concomitant pelvic fracture, overall injury severity, and the operative context of polytrauma care, the present study complements existing multi-institutional evidence and explores this unresolved question.

The primary research question was whether bladder rupture site (EPBR versus IPBR) was associated with hospital length of stay after accounting for concomitant pelvic fracture and overall injury severity. Rupture site was designated as the primary exposure because it is the principal anatomical classification guiding management, whereas concomitant pelvic fracture was examined as a clinically important associated injury and potential confounder. Hospital length of stay was designated as the primary outcome, and ICU length of stay as the secondary outcome. The remaining analyses were considered exploratory or descriptive.

Based on the frequent coexistence of EPBR and pelvic fracture, we expected that patients with EPBR would have longer unadjusted hospital and ICU stays than those with IPBR, but that these differences might be attenuated after accounting for concomitant pelvic fracture and overall injury severity.

## 2. Materials and Methods

### 2.1. Ethics Statement

This retrospective study was conducted in accordance with the Declaration of Helsinki. The study protocol was reviewed and approved by the Institutional Review Board of Gachon University Gil Medical Center (IRB No. GDIRB2026–153; approval date: 30 April 2026). The study period (January 2014–December 2024) represents the historical treatment period reviewed retrospectively. An institutional trauma-registry dataset prepared for submission to the Korean Trauma Data Bank existed as part of routine registry operations; however, research-specific extraction of eligible records, detailed medical-record review, construction of the analytic dataset, and statistical analyses for the present study were initiated only after IRB approval. The requirement for informed consent was waived for the retrospective review of de-identified trauma-registry and medical-record data covering this study period.

### 2.2. Study Design, Setting, and Participants

This was a retrospective cohort study conducted at a single regional trauma center in the Republic of Korea. We screened the institutional trauma registry for patients treated between January 2014 and December 2024 who had an AIS code for bladder injury. The registry dataset had been prepared for submission to the Korean Trauma Data Bank (KTDB), and eligibility and study variables were verified through medical-record review.

Within this registry-based sampling frame, consecutive eligible patients were screened. Repeat hospital encounters were possible, but each individual was included only once on the basis of the index hospitalization for the qualifying traumatic bladder rupture. The 58 screened cases represented 58 unique individuals. Twelve patients were excluded: one patient younger than 18 years, one patient with combined IPBR and EPBR, and ten adult patients with partial laceration, contusion, or hematoma without definite rupture, leaving 46 unique adult patients in the final analysis. Iatrogenic and spontaneous bladder ruptures were outside the eligibility criteria. The patient with combined IPBR and EPBR was excluded because the primary comparison required mutually exclusive rupture-site groups; assigning this mixed injury to either group would have caused exposure misclassification, and a single case could not form a meaningful separate analytical group.

Trauma-registry coding was performed by AIS-trained coordinators and physicians using radiologic findings, operative reports, and final diagnostic records; uncertain cases were resolved through an institutional AIS coding conference. No independent hospital-wide search of discharge diagnoses, radiology reports, or operative records was performed to identify bladder injuries without a registry code.

Definite traumatic bladder rupture and rupture site were established from the contemporaneously documented clinical diagnosis supported by imaging, a urologic examination, or direct intraoperative confirmation. Sex was recorded as a biological variable from the medical records. Race and ethnicity were not analyzed because these variables were not systematically available in the registry.

Radiology reports, operative notes, and contemporaneous medical records were reviewed to characterize the diagnostic and confirmation sequence. Routine trauma CT or CT angiography, additional intravenous contrast-enhanced CT urography, separate retrograde bladder contrast examinations, cystoscopy, and intraoperative confirmation were recorded separately. No patient underwent formal CT cystography with active retrograde bladder filling. Hematuria, perivesical hematoma, and hemoperitoneum were treated as clinical or indirect findings that could raise suspicion but were not considered definitive evidence by themselves. Because the available records did not consistently identify the exact transition from clinical suspicion to definitive confirmation, time to definitive diagnosis and mutually exclusive first-diagnosis categories were not assigned.

### 2.3. Data Collection and Definitions

We collected baseline demographics, trauma type and mechanism, emergency-department vital signs and lactate, Glasgow Coma Scale score, regional AIS scores, standard ISS, bladder AIS full code and post-dot severity, rupture site, concomitant pelvic fracture, associated injuries, diagnostic and confirmation pathway, treatment, operative context, and in-hospital outcomes. Rectal, vaginal, colonic, small-bowel, and mesenteric injuries were recorded as separate non-mutually exclusive binary variables; group comparisons used a binary variable indicating any associated rectal, vaginal, bowel, or mesenteric injury. Core variables used in the primary and secondary analyses were complete for all 46 patients, and no imputation was performed. Treatment-specific variables that did not apply, such as time to repair in nonoperatively managed patients, were recorded as not applicable rather than as missing data.

Injury mechanisms were categorized using trauma-registry data and medical records as traffic accident, fall, slip, struck by person/object, penetrating injury, or unknown. Traffic accidents included motor-vehicle, motorcycle, and pedestrian traffic events. Fall was defined as a fall from height or between levels, whereas slip was defined as a ground-level fall resulting from a slip or trip; these categories were mutually exclusive. Struck by person/object referred to direct external impact by another person or an object without a traffic collision, fall, slip, or penetrating mechanism. The mechanism was classified as unknown when the available documentation was insufficient to assign a specific category.

Data were extracted by one investigator (J.J.) and subsequently reviewed by a second investigator (M.J.J.) for completeness and internal consistency; however, independent duplicate abstraction was not performed. Unclear classifications were resolved through source-record review and consensus with the senior author (K.K.C.). Because exposure, treatment, and outcome variables were retrospectively abstracted from the same source records, the investigators were not blinded to clinical outcomes.

Concomitant pelvic fracture was identified from the institutional trauma registry and radiologic records, primarily from the initial trauma CT or CT angiography; plain pelvic radiographs and operative records were used as supplementary sources when available. The modality in which the fracture was first identified was not recorded as a separate patient-level variable. Pelvic fracture was analyzed as a binary variable indicating its presence or absence. Pelvic-fracture fixation was not collected as a standardized cohort-wide variable. Formal orthopedic classification by fracture type or severity, stable-versus-unstable or open-versus-closed fracture status, fixation technique and timing, and pelvic angioembolization were likewise not consistently available across the entire cohort.

Standard ISS was calculated as the sum of the squares of the highest AIS severity scores in the three most severely injured of the six ISS body regions. To assess the structural contribution of pelvic fracture to ISS, we performed an exploratory sensitivity analysis in which AIS codes for pelvic ring, acetabular, sacral, and iliac fractures were removed while nonpelvic extremity injuries were retained. The maximum AIS severity in each body region was then reassessed, and a modified ISS was recalculated using the same sum-of-squares procedure. This modified score was a nonstandard exploratory sensitivity measure and was not considered a replacement for standard ISS.

Bladder injury AIS full codes were obtained from the institutional trauma registry prepared for submission to the Korean Trauma Data Bank. AIS injury coding was performed according to AIS 2005 Update 2008 by AIS-trained trauma-registry coordinators and physicians. Each full AIS code comprises a pre-dot injury descriptor that specifies the anatomical injury and a post-dot value that ranks its relative severity. Codes were assigned on the basis of documented anatomical findings in radiologic examinations, operative reports, and final diagnostic records. When records were discordant, or the appropriate code was uncertain, the case was reviewed at an institutional AIS coding meeting, and the final code was determined by consensus.

In this cohort, severity 2 primarily represented extraperitoneal bladder-wall lacerations measuring 2 cm or less; severity 3 represented larger extraperitoneal lacerations, intraperitoneal lacerations, or rupture not further specified; and severity 4 represented massive or complex bladder injury, tissue loss, or involvement of the trigone or bladder neck. Seven of the eight severity-2 cases were coded as extraperitoneal bladder-wall lacerations measuring 2 cm or less (540,623.2); the remaining patient had a bladder-injury-not-further-specified registry code (540,699.2), but definite rupture was verified from imaging, operative findings, or contemporaneous medical-record documentation.

Study eligibility was not determined by the post-dot severity value alone; inclusion was restricted to definite traumatic bladder rupture confirmed through imaging, operative findings, or contemporaneous medical-record documentation.

Surgical variables included whether operative bladder repair was performed, surgical approach, repair technique, and the calendar-day interval from emergency-department arrival to initial operative bladder repair; same-day repair was coded as 0 days. For the four nonoperatively managed patients, the repair interval was not applicable, and they were excluded from timing analyses. A bladder-related complication was defined as clinically confirmed persistent or recurrent urinary leakage, including fistulous communication, during the index hospitalization. Leakage was ascertained from follow-up imaging, direct operative findings, or clinically significant urinary drain output with elevated drain-fluid creatinine that prompted additional surgical evaluation or treatment. Limited or inconclusive evaluation alone was not classified as a complication. Because catheter duration and follow-up timing were heterogeneous, no fixed guideline-based duration threshold was applied retrospectively to define a study complication. Reoperation was defined as an additional bladder repair or reinforcement after the initial repair during the index hospitalization; unrelated trauma operations were not included. Mortality was defined separately as all-cause in-hospital mortality and was not considered a bladder-specific outcome.

Operative repair technique was classified as one-layer or two-layer closure according to the description in the operative record. Repairs documented as ‘layer-by-layer’ closure were classified as conventional two-layer closure. Bladder repair was performed by trauma surgeons or urologists, and the closure technique was selected according to surgeon preference and the intraoperative setting rather than a prespecified injury-site protocol. Continuous closure, absorbable sutures, and bladder-filling watertight testing were recorded only when explicitly documented; missing technical details were not inferred. Suprapubic drainage was used selectively when clinically indicated rather than according to a uniform protocol.

For patients with EPBR who underwent operative repair, the primary documented clinical and operative context was categorized as pelvic or orthopedic surgery, bladder-directed repair after preoperative imaging diagnosis, emergency exploratory laparotomy, preperitoneal pelvic packing, PPP gauze removal or exchange, re-exploration prompted by urinary drain findings, internal iliac artery ligation, or perineal open-wound exploration. These categories describe the principal operative context and were not interpreted as mutually exclusive causal indications for surgery.

### 2.4. Statistical Analysis

Descriptive and initial between-group analyses were performed using IBM SPSS Statistics, version 20.0 (IBM Corp., Armonk, NY, USA). Effect estimates, bootstrap confidence intervals, false-discovery-rate adjustment, and exploratory multivariable models were calculated using Python 3.13.14 with NumPy 2.4.4, pandas 3.0.2, and SciPy 1.17.1. Distributional assumptions were examined using descriptive summaries and Shapiro–Wilk tests. Given the small cohort and the non-normal or right-skewed distributions of several variables, continuous variables were summarized as medians with interquartile ranges and compared using the Mann–Whitney U test. The single primary unadjusted comparison was hospital length of stay according to rupture site; ICU length of stay was the secondary outcome, and all remaining group comparisons were exploratory. Pearson’s chi-square test was used for 2 × 2 tables with adequate expected counts, two-sided Fisher exact tests for sparse 2 × 2 tables, and the Fisher–Freeman–Halton exact test for sparse multicategory tables. Effect estimates with 95% CIs are reported as median differences for continuous variables and risk differences for binary variables. Bootstrap CIs were used for median differences and Newcombe CIs for risk differences. Nominal two-sided *p*-values are shown, and Benjamini–Hochberg false-discovery-rate q-values were calculated separately across the 16 prespecified comparisons for each group analysis. Statistical significance was not inferred from exploratory comparisons solely on the basis of a nominal *p* < 0.05. No formal a priori sample-size calculation was performed because all consecutive eligible patients identified during the predefined study period were included. Given the limited sample size, adjusted and subgroup analyses were considered exploratory.

Hospital length of stay was the primary outcome and ICU length of stay the secondary outcome. The primary-outcome analysis evaluated whether rupture site was associated with hospital length of stay after accounting for concomitant pelvic fracture and ISS. Parsimonious exploratory multivariable log-linear models included rupture site (EPBR versus IPBR), concomitant pelvic fracture (yes versus no), and ISS per 10-point increase. Hospital length of stay was modeled as log(days), whereas ICU length of stay was modeled as log(days + 1) because some patients had no ICU stay. HC3 heteroskedasticity-robust standard errors were used, and exponentiated coefficients are reported as adjusted ratios with 95% CIs. The full-cohort models included 46 patients, and a survivor-only sensitivity analysis included 44. Separate adjusted models were not fitted for bladder-related complications, reoperation, or mortality because of the small number of events.

A separate exploratory log-linear model evaluated log(calendar days to initial repair + 1) among the 42 surgically treated patients (21 IPBR and 21 EPBR) using the same three covariates. The four nonoperatively managed patients had no defined repair interval and were excluded. This estimate was interpreted as conditional on operative treatment. De-identified patient-level diagnostic sequences, operative contexts, calendar-day repair intervals, and in-hospital outcomes are provided in Supplementary Table S5.

## 3. Results

### 3.1. Baseline Characteristics

**Figure 1:**
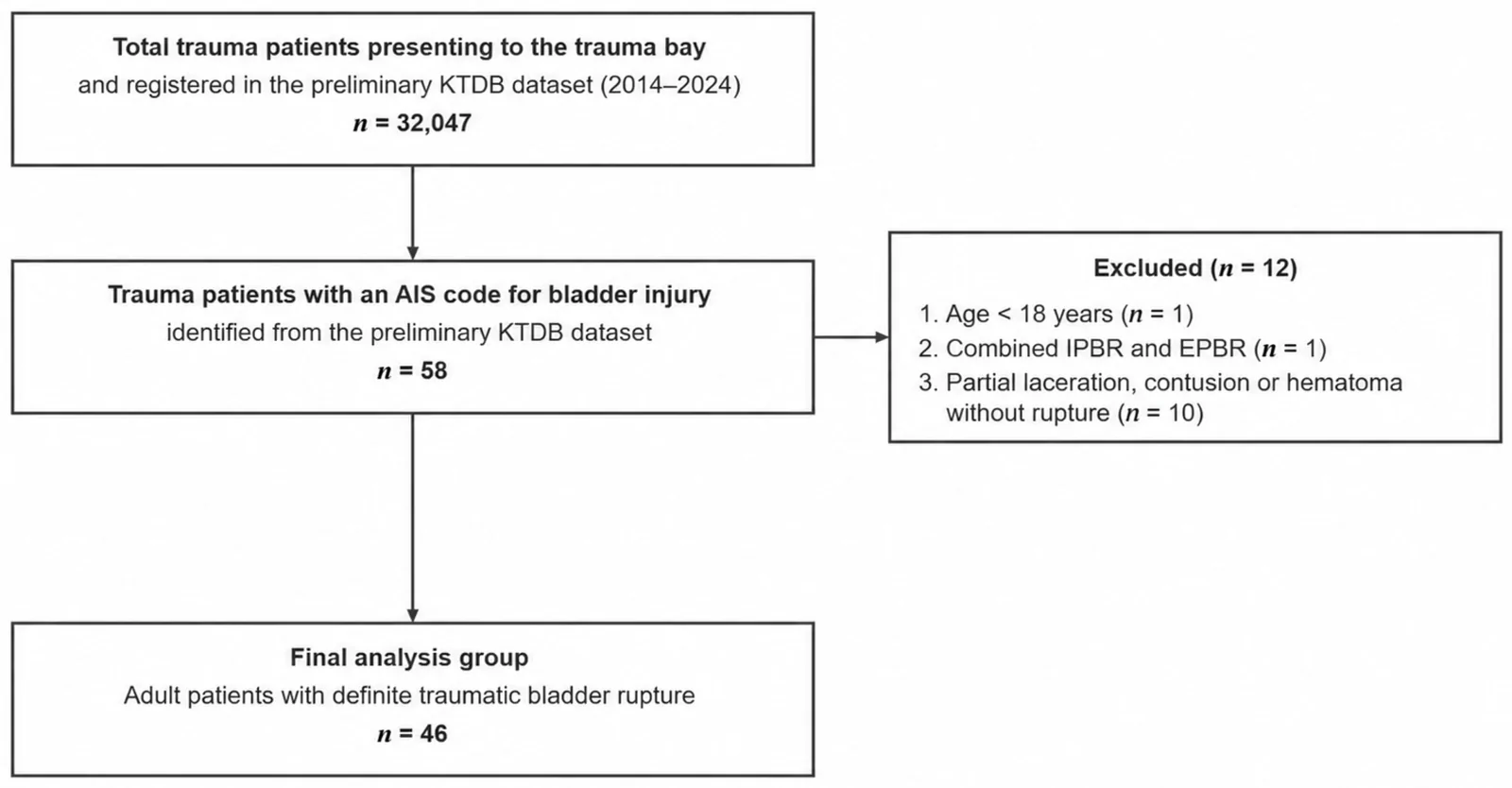
Study flow diagram of patient inclusion. AIS, Abbreviated Injury Scale; EPBR, extraperitoneal bladder rupture; IPBR, intraperitoneal bladder rupture; KTDB, Korean Trauma Data Bank.

Among 58 trauma-registry patients with an AIS code for bladder injury, 46 adults with definite traumatic bladder rupture met the final criteria. The patient-selection process is shown in Figure 1. Core variables used in the primary and secondary analyses were available for all 46 patients. The median age was 50.0 (36.8–60.2) years; 35 patients (76.1%) were male, and 11 (23.9%) were female. Blunt trauma accounted for 44 injuries (95.7%). Traffic accident was the most common mechanism (27, 58.7%), followed by fall (9, 19.6%) and struck-by-person-or-object injury (5, 10.9%) (Table 1).

### 3.2. Injury Characteristics and Management

IPBR was present in 21 patients (45.7%) and EPBR in 25 (54.3%). The median post-dot AIS severity for bladder injury was 3.0 (IQR, 3.0–3.0). AIS severity was 2 in eight patients (17.4%), 3 in 37 (80.4%), and 4 in one (2.2%); the median therefore reflected the predominance of AIS severity 3 in this cohort. Concomitant pelvic fracture was present in 26 patients (56.5%) (Table 2).

The bladder AIS full codes among the 21 patients with IPBR were 540,625.3 in 15 patients (71.4%), 540,624.3 in one (4.8%), and 540,640.3 in five (23.8%); all carried an AIS severity of 3. No patient with IPBR had the AIS severity 4 bladder code 540,626.4. One patient with EPBR and documented trigone/bladder-neck region involvement was coded as 540,626.4.

Any associated rectal, vaginal, bowel, or mesenteric injury was present in nine patients (19.6%). Rectal injury was present in two (4.3%), vaginal injury in one (2.2%), colonic injury in three (6.5%), small-bowel injury in one (2.2%), and mesenteric injury in six (13.0%). Three patients had overlapping injury categories: two had both colonic and mesenteric injuries, and one had rectal, small-bowel, and mesenteric injuries.

All 46 patients (100%) underwent routine trauma CT or CT angiography during initial evaluation. Four (8.7%) underwent additional intravenous contrast-enhanced CT urography. No patient underwent formal CT cystography with active retrograde bladder filling. Separate retrograde bladder contrast examinations were performed in three patients (6.5%): rupture was shown in two, whereas one examination was negative before later operative confirmation. Cystoscopy confirmed rupture in one patient (2.2%). Bladder rupture was directly confirmed intraoperatively in all 42 surgically treated patients, although this was not necessarily the first point of clinical diagnosis. The four nonoperatively managed patients with EPBR were treated with catheter drainage on the basis of the contemporaneous clinical diagnosis and initial trauma CT findings.

Operative bladder repair was performed in 42 patients (91.3%), including all 21 patients with IPBR and 21 of 25 with EPBR. Laparotomy was used in 36 of 42 (85.7%) and laparoscopy in six (14.3%). One-layer closure was used in 12 (28.6%) and two-layer closure in 30 (71.4%). Bladder-related complications occurred in six patients (13.0%); all were urinary-leakage-related events. Persistent leakage was confirmed on follow-up imaging in two patients, including one with rectal fistulous communication; residual leakage was observed during staged abdominal closure in one; and clinically significant urinary drain output with elevated drain-fluid creatinine prompted additional surgical evaluation or treatment in three. A limited or inconclusive examination in one additional patient was not counted as a complication.

Repeat bladder repair was performed in four patients (8.7%) at 2, 5, 8, and 14 days after the initial bladder repair, respectively. The operative circumstances were residual leakage identified during staged abdominal closure with reinforcement of the repair site; cystography-confirmed posterior bladder leakage treated with repeat repair followed by pelvic wound revision; suspected leakage based on urinary drain findings and elevated drain-fluid creatinine treated with revision during the same session as pelvic fixation and wound debridement; and leakage after the initial repair treated with inferior/perineal and anterior bladder-wall repair during the same session as soft-tissue debridement. General trauma operations without additional bladder repair were not classified as reoperations. Two patients (4.3%) died during the index hospitalization: one from acute respiratory distress syndrome 54 days after repair and one from hypoxic brain injury after prehospital cardiac arrest with refractory post-cardiac-arrest circulatory failure 2 days after repair. Neither death was considered directly related to the bladder injury.

### 3.3. Comparison According to Injury Site

In unadjusted comparisons, concomitant pelvic fracture was observed more frequently in patients with EPBR than in those with IPBR (72.0% versus 38.1%; *p* = 0.021; FDR q = 0.078). EPBR was associated with longer hospital stay (median, 44.0 versus 24.0 days; *p* = 0.024; FDR q = 0.078) and ICU stay (8.0 versus 4.0 days; *p* = 0.007; FDR q = 0.037). Among the 42 surgically treated patients, the median calendar-day admission-to-repair interval was 2.0 days for EPBR and 0.0 days for IPBR (*p* < 0.001; FDR q = 0.002). The four nonoperatively managed patients with EPBR were excluded from this timing comparison. Median ISS was 29.0 in EPBR and 19.0 in IPBR (median difference, 10.0; 95% CI, 0.0–24.0; *p* = 0.070; FDR q = 0.186), which did not provide statistically clear evidence of a between-group difference (Table 3).

Among the 21 surgically treated patients with EPBR, the principal documented operative contexts were pelvic or orthopedic surgery in eight (38.1%), bladder-directed repair after preoperative imaging in two (9.5%), emergency exploratory laparotomy in one (4.8%), preperitoneal pelvic packing in one (4.8%), PPP gauze removal or exchange in four (19.0%), re-exploration prompted by urinary drain findings in one (4.8%), internal iliac artery ligation in one (4.8%), and perineal open-wound exploration in three (14.3%). One patient had a bladder bone spicule documented before pelvic open reduction and internal fixation; rectal injury was present in two and vaginal injury in one. The center used a dedicated trauma-surgery and trauma-orthopedic service with daily interdisciplinary conferences and urology participation when needed. These findings describe the operative setting of a selected complex-trauma cohort and do not indicate routine surgery for uncomplicated EPBR.

### 3.4. Comparison According to Pelvic Fracture

In exploratory unadjusted comparisons, standard ISS was higher in patients with concomitant pelvic fracture than in those without pelvic fracture (median, 31.5 versus 10.0; *p* < 0.001; FDR q < 0.001). EPBR was observed more frequently with pelvic fracture (69.2% versus 35.0%; *p* = 0.021; FDR q = 0.067). Patients with pelvic fracture had longer hospital stay (45.5 versus 15.5 days; *p* < 0.001; FDR q < 0.001) and ICU stay (9.5 versus 3.0 days; *p* < 0.001; FDR q < 0.001). Bladder-related complications occurred in six of 26 patients with pelvic fracture and none of 20 without pelvic fracture (*p* = 0.029; FDR q = 0.077) (Table 4).

The largest regional AIS difference was in the extremities/pelvic-girdle region: median AIS was 4 (IQR, 3–4) with pelvic fracture and 0 (IQR, 0–2) without pelvic fracture, and AIS ≥ 3 occurred in 21/26 (80.8%) and 4/20 (20.0%), respectively. Severe chest injury was also numerically more frequent with pelvic fracture [12/26 (46.2%) versus 5/20 (25.0%)], whereas other body regions did not show consistent separation. Regional data were descriptive without separate hypothesis tests. After removal of pelvic-fracture-related AIS codes while retaining nonpelvic extremity injuries, median modified ISS was 18.5 (IQR, 17.0–25.0) with pelvic fracture and 10.0 (IQR, 9.0–19.8) without pelvic fracture (median difference, 8.5; bootstrap 95% CI, −1.0 to 13.0; *p* = 0.014).

### 3.5. Exploratory Multivariable Analyses

In the adjusted primary-outcome analysis, rupture site was not clearly associated with hospital length of stay (EPBR versus IPBR adjusted ratio, 1.02; 95% CI, 0.59–1.76). The estimate for concomitant pelvic fracture was 2.64 (95% CI, 0.84–8.29), and that for ISS per 10-point increase was 1.06 (95% CI, 0.65–1.75). In the secondary adjusted analysis of ICU days + 1, the corresponding estimates were 1.31 (95% CI, 0.77–2.21) for EPBR, 2.36 (95% CI, 0.98–5.72) for pelvic fracture, and 1.34 (95% CI, 0.92–1.97) for ISS per 10 points. These small-sample models were exploratory (Table 5).

Among the 42 surgically treated patients, EPBR was associated with a longer admission-to-repair interval after adjustment for concomitant pelvic fracture and ISS (adjusted ratio for calendar days + 1, 2.21; 95% CI, 1.28–3.82). Estimates for pelvic fracture (1.59; 95% CI, 0.94–2.70) and ISS per 10-point increase (1.00; 95% CI, 0.86–1.16) were not statistically significant. This analysis was conditional on operative treatment and did not include the four nonoperatively managed patients (Table 5).

### 3.6. Exploratory Surgical Subgroup Observations

The operative and nonoperative EPBR groups (*n* = 21 and *n* = 4, respectively) and the laparotomy and laparoscopic IPBR groups (*n* = 15 and *n* = 6, respectively) were small, and the management strategy and surgical approach were determined based on clinical circumstances rather than random assignment. No inferential comparisons were performed; detailed de-identified patient-level characteristics, including the three single-port and three multi-port laparoscopic repairs, are provided in Supplementary Tables S4 and S5.

## 4. Discussion

In this 11-year single-center cohort, rupture site was not clearly associated with the primary outcome of hospital length of stay after adjustment for concomitant pelvic fracture and ISS; the secondary adjusted analysis of ICU length of stay gave a similar result. The unadjusted differences in length of stay therefore appear to reflect, at least in part, the broader trauma context rather than an isolated effect of rupture anatomy. EPBR was observed more frequently with concomitant pelvic fracture, and among surgically treated patients it was associated with a longer calendar-day admission-to-repair interval. The latter result was exploratory and conditional on receipt of operative treatment. The interpretation of EPBR as a clinical marker of complex pelvic trauma remains a hypothesis rather than a causal conclusion.

The high proportion of blunt trauma and the frequent association with pelvic fracture are consistent with previous reports [1,3,4]. The distinction between IPBR and EPBR remains central to management because IPBR generally requires operative repair, whereas uncomplicated EPBR can often be managed with catheter drainage [2,10,11,13]. In this cohort, all patients with IPBR underwent operative bladder repair, whereas four patients with EPBR were managed nonoperatively. Comparisons according to treatment therefore remain vulnerable to nonrandom treatment selection.

The standard ISS difference according to pelvic-fracture status requires particular caution because pelvic fracture directly contributes to the extremities/pelvic-girdle component of ISS. Removing pelvic-fracture-related AIS codes attenuated the between-group difference, although it remained in the exploratory modified-ISS analysis. This nonstandard sensitivity measure suggests that the standard ISS difference was not explained entirely by mathematical inclusion of pelvic fracture, but it does not establish that pelvic fracture independently identifies patients with more severe injuries in other body regions. Accordingly, standard ISS was retained as an overall injury-severity covariate and was not interpreted as independent evidence of extra-pelvic injury burden.

Although uncomplicated EPBR is generally managed with catheter drainage, 21 of 25 patients with EPBR underwent repair. Detailed review showed that repairs often occurred during pelvic or orthopedic surgery, emergency abdominal or hemorrhage-control surgery, staged packing procedures, or other complex trauma operations. A dedicated trauma-surgery and trauma-orthopedic service used daily interdisciplinary conferences, with urologic participation when needed. The high operative proportion therefore describes a selected regional-trauma-center cohort and opportunities for repair during other necessary operations; it should not be interpreted as a general strategy for uncomplicated EPBR or as comparative evidence that operative treatment is superior.

Among the 42 surgically treated patients, EPBR was associated with a longer admission-to-repair interval after adjustment. Unlike IPBR, which was itself an operative indication in this cohort, EPBR repairs occurred across heterogeneous settings, including pelvic or orthopedic surgery, exploratory laparotomy, pelvic packing or gauze removal, and other staged trauma procedures. Preoperative diagnostic certainty also varied. Therefore, the interval most plausibly represents heterogeneous diagnostic, stabilization, and combined-operative pathways. It is not a cohort-wide diagnostic-delay measure, does not include the four nonoperatively managed patients, and should not be interpreted as evidence that EPBR caused delayed treatment.

The diagnostic review highlights an important clinical limitation and implication. Routine trauma CT or CT angiography was universal, whereas formal retrograde CT cystography was not performed and other dedicated bladder examinations were selective. Bladder injuries without a registry code or without definitive follow-up may therefore have been missed, and diagnostic verification was more complete among operatively treated patients. The frequent coexistence of bladder rupture and pelvic fracture should heighten clinical awareness and reinforce consideration of guideline-concordant active retrograde cystographic evaluation when bladder injury is clinically suspected [2,8,10,11]; however, the present study did not test whether a structured early-evaluation protocol improves outcomes. In MiGUTS, concomitant bladder-neck or urethral injury was the only independent predictor of bladder-related complications, whereas a recent pelvic-fracture cohort described delayed lower urinary tract injury diagnoses, often after initial prioritization of life-saving care [6,12].

Management should consider both bladder rupture site and the overall clinical context. IPBR generally requires operative repair, whereas uncomplicated EPBR may be managed with catheter drainage [2,10,11,13]. In patients with EPBR, overall injury severity, hemodynamic status, associated injuries, persistent urinary extravasation, and planned pelvic fixation should also be considered. When EPBR coexists with a pelvic fracture requiring internal fixation, retrospective evidence may inform individualized management but does not establish the superiority of operative repair [12,14,15]. Early coordination among trauma, urologic, and orthopedic teams may help align diagnostic evaluation and individualized treatment planning.

The operative and nonoperative EPBR groups and the laparoscopic subgroups were very small and selected nonrandomly. Their patient-level characteristics are therefore presented to improve transparency, not to estimate comparative effectiveness or safety. Observed differences may primarily reflect injury burden, hemodynamic status, associated procedures, surgeon selection, and other unmeasured clinical factors.

This study has several limitations. First, its retrospective single-center design limits generalizability and creates risks of selection, information, and verification bias. The cohort was ascertained through trauma-registry AIS codes, and no independent hospital-wide search was performed for uncoded cases; changes in coding, imaging, surgical experience, and treatment practices across the 11-year period may also have affected case ascertainment and management. Exclusion of the single patient with combined IPBR and EPBR was necessary for the mutually exclusive rupture-site comparison but may limit generalizability to mixed rupture patterns.

Second, data abstraction was performed by a single investigator who was not blinded to clinical outcomes; reviewer-dependent misclassification cannot be excluded, although ambiguous cases were re-reviewed and resolved through consensus with the senior author and study team.

Third, formal retrograde CT cystography was not routine; additional bladder-directed examinations were selective, standardized fill volumes and complete urethral-assessment data were unavailable, and hematuria was not consistently recorded. Some bladder injuries may therefore have been missed or misclassified, and direct operative confirmation was necessarily more common in the surgical group.

Fourth, detailed orthopedic classification of pelvic fractures and cohort-wide data regarding pelvic fracture fixation, open-fracture status, and pelvic angioembolization were not collected as standardized variables.

Fifth, detailed operative variables, including continuous versus interrupted closure, suture material, and watertight testing, were not uniformly documented, and Foley catheter duration was incompletely available. Post-treatment imaging and urologic follow-up modalities were reviewed case by case, but their timing and modality were heterogeneous and did not follow a uniform protocol; dedicated cystography immediately before catheter removal was not performed in every patient. Therefore, the observed follow-up modalities should not be interpreted as a standardized cystography protocol or uniform time to healing.

Sixth, the sample was small, especially for complications, repeat repair, mortality, and treatment subgroups; the numerous exploratory comparisons increase the possibility of false-positive findings despite FDR reporting; survivor bias may affect length-of-stay comparisons, and indication bias and treatment-selection bias are substantial in the operative subgroup analyses.

Seventh, a single reliable numerical time to definitive diagnosis could not be reconstructed, hourly bladder-specific operative timestamps were unavailable for many combined operations, and the admission-to-repair interval could not be separated consistently into diagnosis-to-repair and stabilization or scheduling components.

Finally, despite exploratory adjustment, residual confounding by physiology, associated injuries, treatment selection, operative plans, and surgeon preference remains likely. Outcomes were limited to the index hospitalization because complete healing time, antibiotic use, and long-term post-discharge outcomes were not consistently available.

## 5. Conclusions

After adjustment for concomitant pelvic fracture and ISS, rupture site was not clearly associated with hospital or ICU length of stay. Among surgically treated patients, EPBR was associated with a longer admission-to-repair interval; this exploratory finding was conditional on operative treatment and may reflect complex trauma-care pathways rather than diagnostic delay. The frequent coexistence of bladder rupture and pelvic fracture reinforces the clinical importance of careful bladder assessment in patients with severe pelvic trauma.

## Figures and Tables

**Table 1:** Baseline characteristics of patients with traumatic bladder rupture.

Variable	Category	*n* (%) or Median (IQR)
Age, years		50.0 (36.8–60.2)
Sex	Male	35 (76.1%)
	Female	11 (23.9%)
Injury type	Blunt	44 (95.7%)
	Penetrating	2 (4.3%)
Injury mechanism	Traffic accident	27 (58.7%)
	Fall	9 (19.6%)
	Slip	2 (4.3%)
	Struck by person/object	5 (10.9%)
	Penetrating	2 (4.3%)
	Unknown	1 (2.2%)
Route of presentation	Direct admission	27 (58.7%)
	Transfer from another hospital	19 (41.3%)
Systolic blood pressure, mmHg	total	118.0 (99.8–144.2)
	≤90 mmHg	6 (13.0%)
Heart rate, beats/min		101.0 (80.5–120.8)
Glasgow Coma Scale score		15.0 (13.5–15.0)
Lactate, mmol/L		3.4 (2.4–5.1)

Values are presented as median (interquartile range) or *n* (%). Data were available for all 46 patients.

**Table 2:** Injury characteristics, management, and clinical outcomes.

Variable	Category	*n* (%) or Median (IQR)
**Injury Characteristics**		
Injury site	Intraperitoneal	21 (45.7%)
	Extraperitoneal	25 (54.3%)
Bladder AIS post-dot severity	total	3.0 (3.0–3.0)
	2	8 (17.4%)
	3	37 (80.4%)
	4	1 (2.2%)
Injury Severity Score		22.0 (10.0–34.0)
Associated injury	Rectal	2 (4.3%)
	Vaginal	1 (2.2%)
	Colonic	3 (6.5%)
	Small-bowel	1 (2.2%)
	Mesenteric	6 (13.0%)
	Any associated rectal, vaginal, bowel, or mesenteric injury	9 (19.6%)
Concomitant pelvic fracture		26 (56.5%)
**Diagnostic and Management Characteristics**		
Diagnostic/confirmation modality	Routine trauma CT or CT angiography	46 (100%)
	Additional intravenous contrast-enhanced CT urography	4 (8.7%)
	Formal CT cystography with active retrograde filling	0 (0.0%)
	Separate retrograde bladder contrast examination	3 (6.5%)
	Cystoscopy	1 (2.2%)
	Direct intraoperative confirmation	42 (91.3%)
Operative bladder repair		42 (91.3%)
Surgical approach ^†^	Laparotomy	36 (85.7%)
	Laparoscopy	6 (14.3%)
Repair technique ^†^	One-layer closure	12 (28.6%)
	Two-layer closure	30 (71.4%)
Principal operative setting for EPBR repair ^‡^	Pelvic or orthopedic surgery	8 (38.1%)
	Bladder-directed repair after preoperative imaging diagnosis	2 (9.5%)
	Emergency exploratory laparotomy	1 (4.8%)
	Preperitoneal pelvic packing	1 (4.8%)
	PPP gauze removal or exchange	4 (19.0%)
	Re-exploration prompted by urinary drain findings	1 (4.8%)
	Internal iliac artery ligation	1 (4.8%)
	Perineal open-wound exploration	3 (14.3%)
Admission-to-initial-operative-bladder-repair interval ^§^, calendar days		1.0 (0.0–3.0)
**In-Hospital Outcomes**		
Bladder-related complication	Total	6 (13.0%)
	Identified on follow-up imaging	2 (4.3%)
	Identified during staged abdominal closure	1 (2.2%)
	Drain findings prompting additional operative evaluation or treatment	3 (6.5%)
Repeat bladder repair		4 (8.7%)
Hospital stay, days		27.5 (16.0–58.0)
ICU stay, days		6.0 (3.0–12.8)
All-cause in-hospital mortality		2 (4.3%)

Values are presented as median (interquartile range) or *n* (%). Associated injury subtypes and diagnostic/confirmation modalities were not mutually exclusive. ^†^ Percentages were calculated among patients who underwent operative bladder repair (*n* = 42). ^‡^ Percentages were calculated among surgically treated patients with EPBR (*n* = 21). ^§^ The admission-to-initial-operative-bladder-repair interval was calculated among surgically treated patients (*n* = 42). AIS, Abbreviated Injury Scale; CT, computed tomography; EPBR, extraperitoneal bladder rupture; ICU, intensive care unit; PPP, preperitoneal pelvic packing.

**Table 3:** Comparison of clinical characteristics and outcomes according to bladder rupture site.

Variable	Category	IPBR (*n* = 21)	EPBR (*n* = 25)	*p*-Value	Effect Estimate (95% CI)	FDR q
Age, years		50.0 (34.5–59.0)	50.0 (41.0–61.0)	0.354	Median difference 0.0 (95% CI −10.0 to 21.0)	0.540
Sex	Male	17 (81.0%)	18 (72.0%)			
	Female	4 (19.0%)	7 (28.0%)	0.478	Risk difference 9.0% (95% CI −16.1% to 31.6%)	0.638
Injury mechanism	Traffic accident	13 (61.9%)	14 (56.0%)	0.169	No single effect estimate	—
	Fall	2 (9.5%)	7 (28.0%)		—	—
	Slip	2 (9.5%)	0 (0.0%)		—	—
	Struck by person/object	3 (14.3%)	2 (8.0%)		—	—
	Penetrating injury	0 (0.0%)	2 (8.0%)		—	—
	Unknown	1 (4.8%)	0 (0.0%)		—	—
Route of presentation	Direct admission	14 (66.7%)	13 (52.0%)		—	—
	Transfer from another hospital	7 (33.3%)	12 (48.0%)	0.377	Risk difference 14.7% (95% CI −13.2% to 39.2%)	—
Glasgow Coma Scale score		15.0 (13.5–15.0)	15.0 (13.0–15.0)	0.371	Median difference 0.0 (95% CI −1.0 to 0.0)	0.540
Systolic blood pressure, mmHg	total	135.0 (100.5–146.5)	111.0 (99.5–131.0)	0.137	Median difference −24.0 (95% CI −38.0 to 11.0)	0.273
	≤90 mmHg	2 (9.5%)	4 (16.0%)	0.673	Risk difference 6.5% (95% CI −15.2% to 26.4%)	0.720
Lactate, mmol/L		3.40 (2.85–4.85)	2.80 (2.10–7.25)	0.675	Median difference −0.6 (95% CI −1.7 to 1.6)	0.720
Any associated rectal, vaginal, bowel, or mesenteric injury		4 (19.0%)	5 (20.0%)	1.000	Risk difference 1.0% (95% CI −22.8% to 23.2%)	1.000
Concomitant pelvic fracture		8 (38.1%)	18 (72.0%)	0.021	Risk difference 33.9% (95% CI 5.2% to 56.0%)	0.078
Bladder AIS post-dot severity	2	0 (0.0%)	8 (32.0%)	0.003	No single effect estimate	0.021
	3	21 (100.0%)	16 (64.0%)		—	—
	4	0 (0.0%)	1 (4.0%)		—	—
Injury Severity Score		19.0 (9.5–24.5)	29.0 (17.0–35.5)	0.070	Median difference 10.0 (95% CI 0.0 to 24.0)	0.186
Maximum AIS by ISS body region						
Head or neck	Median (IQR)	0 (0–0)	0 (0–0)	—	—	—
	AIS ≥ 3, *n* (%)	2 (9.5%)	3 (12.0%)	—	—	—
Face	Median (IQR)	0 (0–0)	0 (0–0)	—	—	—
	AIS ≥ 3, *n* (%)	0 (0.0%)	1 (4.0%)	—	—	—
Chest	Median (IQR)	0 (0–3)	0 (0–3)	—	—	—
	AIS ≥ 3, *n* (%)	7 (33.3%)	10 (40.0%)	—	—	—
Abdominal or pelvic content	Median (IQR)	3 (3–3)	3 (3–3)	—	—	—
	AIS ≥ 3, *n* (%)	21 (100.0%)	19 (76.0%)	—	—	—
Extremities or pelvic girdle	Median (IQR)	2 (0–3)	4 (2–4)	—	—	—
	AIS ≥ 3, *n* (%)	8 (38.1%)	17 (68.0%)	—	—	—
External	Median (IQR)	0 (0–1)	0 (0–1)	—	—	—
	AIS ≥ 3, *n* (%)	0 (0.0%)	0 (0.0%)	—	—	—
Operative bladder repair		21 (100.0%)	21 (84.0%)	0.114	Risk difference −16.0% (95% CI −34.7% to 2.2%)	0.261
Operative approach among repaired patients ^†^	Laparotomy	15 (71.4%)	21 (100.0%)	—	—	—
	Laparoscopy	6 (28.6%)	0 (0.0%)	—	—	—
Closure method among repaired patients ^†^	One-layer closure	5 (23.8%)	7 (33.3%)	—	—	—
	Two-layer closure	16 (76.2%)	14 (66.7%)	—	—	—
Admission-to-initial-operative-bladder-repair interval *, calendar days		0.0 (0.0–1.0)	2.0 (1.0–6.5)	<0.001	Median difference 2.0 (95% CI 1.0 to 6.0)	0.002
ICU stay, days		4.0 (3.0–7.5)	8.0 (4.5–21.5)	0.007	Median difference 4.0 (95% CI 1.0 to 12.0)	0.037
Hospital stay, days		24.0 (14.5–29.0)	44.0 (26.0–65.5)	0.024	Median difference 20.0 (95% CI 1.0 to 41.0)	0.078
Bladder-related complication		1 (4.8%)	5 (20.0%)	0.198	Risk difference 15.2% (95% CI −5.9% to 34.8%)	0.352
Reoperation		1 (4.8%)	3 (12.0%)	0.614	Risk difference 7.2% (95% CI −12.3% to 25.6%)	0.720
All-cause in-hospital mortality		0 (0.0%)	2 (8.0%)	—	—	—

Values are median (interquartile range) or *n* (%). Except for the prespecified primary hospital-stay comparison and secondary ICU-stay comparison, the between-group tests are unadjusted exploratory comparisons. Effect estimates are EPBR minus IPBR and are median differences with bootstrap 95% CIs for continuous variables or risk differences with Newcombe 95% CIs for binary variables. Nominal *p*-values are from Mann–Whitney U, Pearson chi-square, two-sided Fisher exact, or Fisher–Freeman–Halton exact tests as appropriate. FDR q-values use the Benjamini–Hochberg procedure across the 16 prespecified comparisons in this table. Female sex and concomitant pelvic fracture were analyzed with Pearson chi-square tests; systolic blood pressure ≤ 90 mmHg, operative bladder repair, bladder-related complication, and reoperation were analyzed with two-sided Fisher exact tests; bladder AIS severity was analyzed with the Fisher–Freeman–Halton exact test. Associated injury represents any associated rectal, vaginal, bowel, or mesenteric injury and was analyzed with a two-sided Fisher exact test. Mortality is descriptive without hypothesis testing. * The admission-to-initial-operative-bladder-repair interval was measured in calendar days among 21 patients with IPBR and 21 surgically treated patients with EPBR; four nonoperatively managed patients with EPBR were not applicable and were excluded. ^†^ Operative-approach and closure-method percentages were calculated among patients who underwent operative bladder repair (IPBR, *n* = 21; EPBR, *n* = 21) and were summarized descriptively without hypothesis testing. Trauma mechanism and route of presentation were not included in the 16-comparison FDR family. Maximum AIS values by ISS body region and operative-technique distributions were summarized descriptively without hypothesis testing, effect estimation, or FDR adjustment. Trauma mechanism used the Fisher–Freeman–Halton exact test; route of presentation used a two-sided Fisher exact test. AIS, Abbreviated Injury Scale; CI, confidence interval; EPBR, extraperitoneal bladder rupture; FDR, false discovery rate; ICU, intensive care unit; IPBR, intraperitoneal bladder rupture; ISS, Injury Severity Score.

**Table 4:** Comparison of clinical characteristics and outcomes according to concomitant pelvic fracture.

Variable	Category	With Pelvic Fracture (*n* = 26)	Without Pelvic Fracture (*n* = 20)	*p*-Value	Effect Estimate (95% CI)	FDR q
Age, years		46.5 (32.5–61.0)	50.0 (42.2–58.5)	0.579	Median difference −3.5 (95% CI −16.0 to 10.0)	0.713
Sex	Male	20 (76.9%)	15 (75.0%)			
	Female	6 (23.1%)	5 (25.0%)	1.000	Risk difference −1.9% (95% CI −26.9% to 21.5%)	1.000
Glasgow Coma Scale score		15.0 (12.0–15.0)	15.0 (15.0–15.0)	0.295	Median difference 0.0 (95% CI −1.0 to 0.0)	0.441
Systolic blood pressure, mmHg	total	109.0 (96.5–132.8)	134.0 (107.0–150.8)	0.094	Median difference −25.0 (95% CI −42.5 to 12.0)	0.215
	≤90 mmHg	4 (15.4%)	2 (10.0%)	0.684	Risk difference 5.4% (95% CI −16.7% to 24.9%)	0.781
Lactate, mmol/L		3.30 (2.38–4.97)	3.55 (2.32–5.17)	0.799	Median difference −0.2 (95% CI −2.0 to 1.0)	0.852
Injury site	Intraperitoneal	8 (30.8%)	13 (65.0%)			
	Extraperitoneal	18 (69.2%)	7 (35.0%)	0.021	Risk difference 34.2% (95% CI 5.2% to 56.3%)	0.067
Any associated rectal, vaginal, bowel, or mesenteric injury		7 (26.9%)	2 (10.0%)	0.262	Risk difference 16.9% (95% CI −7.1% to 37.4%)	0.441
Bladder AIS post-dot severity	2	3 (11.5%)	5 (25.0%)	0.435	No single effect estimate	0.580
	3	22 (84.6%)	15 (75.0%)		—	—
	4	1 (3.8%)	0 (0.0%)		—	—
Injury Severity Score		31.5 (21.5–36.5)	10.0 (9.0–21.2)	< 0.001	Median difference 21.5 (95% CI 10.5 to 25.0)	<0.001
Maximum AIS by ISS body region						
Head or neck	Median (IQR)	0 (0–0)	0 (0–0)	—	—	—
	AIS ≥ 3, *n* (%)	3 (11.5%)	2 (10.0%)	—	—	—
Face	Median (IQR)	0 (0–0)	0 (0–0)	—	—	—
	AIS ≥ 3, *n* (%)	0 (0.0%)	1 (5.0%)	—	—	—
Chest	Median (IQR)	2 (0–3)	0 (0–2.3)	—	—	—
	AIS ≥ 3, *n* (%)	12 (46.2%)	5 (25.0%)	—	—	—
Abdominal or pelvic contents	Median (IQR)	3 (3–3)	3 (3–3)	—	—	—
	AIS ≥ 3, *n* (%)	24 (92.3%)	16 (80.0%)	—	—	—
Extremities or pelvic girdle	Median (IQR)	4 (3–4)	0 (0–2)	—	—	—
	AIS ≥ 3, *n* (%)	21 (80.8%)	4 (20.0%)	—	—	—
External	Median (IQR)	0 (0–1)	0.5 (0–1)	—	—	—
	AIS ≥ 3, *n* (%)	0 (0.0%)	0 (0.0%)	—	—	—
Modified ISS excluding pelvic-fracture AIS codes		18.5 (17.0–25.0)	10.0 (9.0–19.8)	0.014	Median difference 8.5 (95% CI −1.0 to 13.0)	—
Operative bladder repair		25 (96.2%)	17 (85.0%)	0.303	Risk difference 11.2% (95% CI −6.8% to 32.4%)	0.441
Admission-to-initial-operative-bladder-repair interval *, calendar days		2.0 (1.0–6.0)	0.0 (0.0–1.0)	0.002	Median difference 2.0 (95% CI 1.0 to 4.0)	0.007
ICU stay, days		9.5 (6.0–24.5)	3.0 (0.0–4.8)	<0.001	Median difference 6.5 (95% CI 4.0 to 14.5)	<0.001
Hospital stay, days		45.5 (27.0–66.2)	15.5 (10.5–26.8)	<0.001	Median difference 30.0 (95% CI 9.0 to 48.5)	<0.001
Bladder-related complication		6 (23.1%)	0 (0.0%)	0.029	Risk difference 23.1% (95% CI 3.0% to 42.1%)	0.077
Reoperation		4 (15.4%)	0 (0.0%)	0.121	Risk difference 15.4% (95% CI −3.2% to 33.5%)	0.243
All-cause in-hospital mortality		1 (3.8%)	1 (5.0%)	—	—	—

Values are median (interquartile range) or *n* (%). All between-group tests in this table are unadjusted exploratory comparisons. Effect estimates are pelvic-fracture minus no-pelvic-fracture values and are median differences with bootstrap 95% CIs for continuous variables or risk differences with Newcombe 95% CIs for binary variables. Nominal *p*-values are from Mann–Whitney U, Pearson chi-square, two-sided Fisher exact, or Fisher–Freeman–Halton exact tests as appropriate. FDR q-values use the Benjamini–Hochberg procedure across the 16 prespecified comparisons in this table. Injury site used Pearson chi-square; female sex, systolic blood pressure ≤ 90 mmHg, associated injury, operative bladder repair, bladder-related complication, and reoperation used two-sided Fisher exact tests; bladder AIS severity used the Fisher–Freeman–Halton exact test. Maximum AIS values by ISS body region were summarized descriptively without separate hypothesis testing, effect estimation, or FDR adjustment. Modified ISS excluding pelvic-fracture-related AIS codes is a nonstandard exploratory sensitivity score; nonpelvic extremity codes were retained, and the displayed *p*-value was not included in the table-wide FDR family. Mortality is descriptive without hypothesis testing. * The admission-to-initial-operative-bladder-repair interval was measured in calendar days among 42 patients who underwent operative bladder repair (25 with and 17 without concomitant pelvic fracture); nonoperatively managed patients were excluded from this analysis. AIS, Abbreviated Injury Scale; CI, confidence interval; FDR, false discovery rate; ICU, intensive care unit; ISS, Injury Severity Score.

**Table 5:** Exploratory multivariable analyses of hospital length of stay, ICU length of stay, and admission-to-repair interval.

Analysis	Variable	Hospital Stay Adjusted Ratio (95% CI)	ICU Days + 1 Adjusted Ratio (95% CI)	Admission-to-Repair Interval Days + 1 Adjusted Ratio (95% CI)
Full cohort (*n* = 46)	EPBR vs. IPBR	1.02 (0.59–1.76)	1.31 (0.77–2.21)	—
	Pelvic fracture	2.64 (0.84–8.29)	2.36 (0.98–5.72)	—
	ISS per 10 points	1.06 (0.65–1.75)	1.34 (0.92–1.97)	—
Survivors only (*n* = 44)	EPBR vs. IPBR	1.15 (0.74–1.76)	1.37 (0.83–2.26)	—
	Pelvic fracture	1.56 (0.94–2.58)	1.68 (0.92–3.05)	—
	ISS per 10 points	1.35 (1.08–1.69)	1.54 (1.16–2.04)	—
Surgically treated patients (*n* = 42)	EPBR vs. IPBR	—	—	2.21 (1.28–3.82)
	Pelvic fracture	—	—	1.59 (0.94–2.70)
	ISS per 10 points	—	—	1.00 (0.86–1.16)

Hospital length of stay was the primary outcome; ICU length of stay was secondary. Each exploratory model simultaneously included rupture site, concomitant pelvic fracture, and ISS. Hospital-stay and ICU-stay models used the full cohort (*n* = 46), with a survivor-only sensitivity analysis (*n* = 44). The admission-to-repair model was restricted to the 42 surgically treated patients and is conditional on operative treatment. Adjusted ratios are exponentiated coefficients from the log-linear models and represent multiplicative ratios in in-hospital length of stay, ICU days + 1, or admission-to-repair days + 1, as applicable. HC3 heteroskedasticity-robust standard errors were used to calculate 95% CIs. CI, confidence interval; EPBR, extraperitoneal bladder rupture; HC3, heteroskedasticity-consistent type 3; ICU, intensive care unit; IPBR, intraperitoneal bladder rupture; ISS, Injury Severity Score.

## Data Availability

Restrictions apply to the availability of the full patient-level dataset because of institutional and privacy considerations. De-identified patient-level data supporting the reported descriptive results are included in Supplementary Tables S4 and S5. Requests for additional de-identified data may be directed to the corresponding author and will be considered subject to institutional approval, applicable privacy restrictions, and an appropriate data-use agreement.

## Supplementary Materials

The following supporting information can be downloaded at: https://www.mdpi.com/article/10.3390/jcm15187072/s1, Table S1. Comparison of surgical repair and nonoperative management in patients with extraperitoneal bladder rupture; Table S2. Comparison of laparotomy and laparoscopy in patients with intraperitoneal bladder rupture; Table S3. Descriptive comparison of single-port and multi-port laparoscopy in patients with intraperitoneal bladder rupture; Table S4: De-identified patient-level characteristics and treatment pathways of four nonoperatively managed EPBR patients and six laparoscopically repaired IPBR patients; Table S5: De-identified patient-level characteristics, diagnostic and confirmation pathways, treatment, calendar-day repair interval, and in-hospital outcomes of all 46 patients.

## Abbreviations

The following abbreviations are used in this manuscript:

AIS	Abbreviated Injury Scale
CI	Confidence interval
CT	Computed tomography
EPBR	Extraperitoneal bladder rupture
FDR	False discovery rate
HC3	Heteroskedasticity-consistent type 3
ICU	Intensive care unit
IPBR	Intraperitoneal bladder rupture
IQR	Interquartile range
IRB	Institutional Review Board
ISS	Injury Severity Score
KTDB	Korean Trauma Data Bank
PPP	Preperitoneal pelvic packing
SBP	Systolic blood pressure
