# Supplementary material for: Clinical Characteristics and In-Hospital Outcomes of Traumatic Bladder Rupture: An 11-Year Retrospective Cohort Study at a Single Regional Trauma Center
Source: J Clin Med. 2026 Sep 11;15(18):7072. doi: 10.3390/jcm15187072 (PMC13607321; doi:10.3390/jcm15187072)
Supplement: Supplementary file 1 [file jcm-15-07072-s001.zip › Supplementary Tables S1-S3.pdf]

Supplementary Tables: Clinical Outcomes of Traumatic Bladder Rupture

Table S1. Comparison of surgical repair and nonoperative management in patients with extraperitoneal bladder rupture.

Variable	Surgical repair (n = 21)	Nonoperative management (n = 4)	P-value
Age, years	57.0 (39.0-62.0)	48.5 (44.2-52.8)	0.629
Injury Severity Score	29.0 (19.5-39.5)	8.5 (5.0-27.8)	0.048
Hospital stay, days	45.0 (27.0-66.5)	9.5 (4.8-46.5)	0.049
ICU stay, days	10.0 (6.5-26.0)	1.0 (0.0-3.5)	0.003
Male sex	14 (66.7%)	4 (100.0%)	0.294
Concomitant pelvic fracture	17 (81.0%)	1 (25.0%)	0.053
One-layer suture	7 (33.3%)	Not applicable	
Bladder-related complication	6 (28.6%)	0 (0.0%)	0.540
Reoperation	3 (14.3%)	0 (0.0%)	1.000

Values are presented as median (interquartile range) or n (%). P-values are two-sided and are from the Mann-Whitney U test or Fisher exact test. One-layer suture is not applicable to the nonoperative group.

Table S2. Comparison of laparotomy and laparoscopy in patients with intraperitoneal bladder rupture.

Variable	Laparotomy (n = 15)	Laparoscopy (n = 6)	P-value
Age, years	50.0 (36.0-59.0)	35.5 (22.5-59.2)	0.242
Injury Severity Score	18.0 (9.0-22.0)	22.0 (15.2-28.8)	0.270
Hospital stay, days	25.0 (13.0-31.0)	22.0 (16.0-27.8)	0.938
ICU stay, days	4.0 (3.0-7.0)	4.0 (2.2-8.5)	0.875
Male sex	13 (86.7%)	4 (66.7%)	0.544
Concomitant pelvic fracture	5 (33.3%)	3 (50.0%)	0.631
One-layer suture	1 (6.7%)	4 (66.7%)	0.011
Bladder-related complication	1 (6.7%)	0 (0.0%)	1.000
Reoperation	1 (6.7%)	0 (0.0%)	1.000

Values are presented as median (interquartile range) or n (%). P-values are two-sided and are from the Mann-Whitney U test or Fisher exact test.

Table S3. Descriptive comparison of single-port and multi-port laparoscopy in patients with intraperitoneal bladder rupture.

Variable	Single-port laparoscopy (n = 3)	Multi-port laparoscopy (n = 3)
Age, years	34.0 (21.0-60.0)	37.0 (23.0-59.0)
Injury Severity Score	22.0 (22.0-34.0)	17.0 (10.0-27.0)
Hospital stay, days	17.0 (13.0-24.0)	27.0 (20.0-30.0)
ICU stay, days	4.0 (3.0-8.0)	4.0 (0.0-10.0)
Male sex	3 (100.0%)	1 (33.3%)
Concomitant pelvic fracture	1 (33.3%)	2 (66.7%)
One-layer suture	3 (100.0%)	1 (33.3%)
Bladder-related complication	0 (0.0%)	0 (0.0%)
Reoperation	0 (0.0%)	0 (0.0%)

Values are presented as median (interquartile range) or n (%). No statistical testing was performed because each group contained only three patients.
