# Supplementary material for: Clinical Characteristics and In-Hospital Outcomes of Traumatic Bladder Rupture: An 11-Year Retrospective Cohort Study at a Single Regional Trauma Center
Source: J Clin Med. 2026 Sep 11;15(18):7072. doi: 10.3390/jcm15187072 (PMC13607321; doi:10.3390/jcm15187072)
Supplement: Supplementary file 1 [file jcm-15-07072-s001.zip › Supplementary Tables S4 and S5.pdf]

Supplementary Materials

Clinical characteristics and in-hospital outcomes of traumatic bladder rupture: an 11-year retrospective cohort study at a single regional trauma center

Scope. This supplement provides de-identified patient-level data for the very small clinical subgroups (Table S4) and for all 46 unique patients in the full cohort (Table S5). Names, medical-record numbers, and exact calendar dates are excluded. The subgroup data are descriptive and are not intended to support comparative-effectiveness or safety inference.

Definitions. AIS values are post-dot severity scores from AIS 2005 Update 2008. Same-day operative bladder repair is coded as 0 calendar days; the interval is not applicable to nonoperatively managed patients. Bladder-related complication, repeat bladder repair, and all-cause mortality refer to events during the index hospitalization.

Supplementary Table S4

De-identified patient-level characteristics and treatment pathways of four nonoperatively managed EPBR patients and six laparoscopically repaired IPBR patients.

Table S4 — Panel A. Injury characteristics.

Ten patients; descriptive presentation only.

Study ID	Rupture site	Trauma type	Trauma mechanism	Pelvic fracture	Bladder AIS post-dot severity	Associated rectal/vaginal/bowel/mesenteric injury	Regional AIS profile
1	IPBR	Blunt	Fall from height	No	3	—	Chest AIS 3; Abdomen AIS 3; Pelvis/extremities AIS 2
11	IPBR	Blunt	Fall from height	Yes	3	—	Chest AIS 2; Abdomen AIS 3; Pelvis/extremities AIS 2; External AIS 1
13	IPBR	Blunt	Ground-level fall	No	3	—	Abdomen AIS 3; External AIS 1
16	IPBR	Blunt	Traffic accident	Yes	3	—	Face AIS 1; Chest AIS 3; Abdomen AIS 3; Pelvis/extremities AIS 3; External AIS 1
17	IPBR	Blunt	Traffic accident	No	3	—	Head/neck AIS 3; Face AIS 2; Abdomen AIS 3; Pelvis/extremities AIS 2; External AIS 1
20	EPBR	Blunt	Traffic accident	No	2	—	Chest AIS 2; Abdomen AIS 2
24	EPBR	Blunt	Traffic accident	Yes	2	—	Chest AIS 3; Abdomen AIS 3; Pelvis/extremities AIS 4
26	EPBR	Blunt	Struck by blunt object	No	3	—	Abdomen AIS 3
27	IPBR	Blunt	Traffic accident	Yes	3	Colonic and mesenteric injuries	Chest AIS 3; Abdomen AIS 4; Pelvis/extremities AIS 3
31	EPBR	Blunt	Fall from height	No	2	—	Abdomen AIS 2

Table S4 — Panel B. Diagnostic pathways, treatment, and in-hospital outcomes.

Rows correspond to the Study IDs in Panel A.

Study ID	Diagnostic/confirmation pathway and timing	Treatment	Principal operative setting	Calendar days to initial operative bladder repair	Bladder-related complication	Repeat bladder repair	All-cause in-hospital mortality
1	Day 0 trauma CT: IPBR; Day 2 laparoscopic repair/direct confirmation.	Laparoscopic bladder repair (single-port)	Operative repair undertaken for IPBR; direct anatomical confirmation during repair.	2	No confirmed bladder-related complication	No repeat bladder repair	No in-hospital death
11	Day 0 trauma CT: suspected bladder rupture; day 3 laparoscopic repair/direct confirmation.	Laparoscopic bladder repair (multi-port)	Operative repair undertaken for IPBR; direct anatomical confirmation during repair.	3	No confirmed bladder-related complication	No repeat bladder repair	No in-hospital death
13	Pre-admission outside abdominopelvic CT: hemoperitoneum and probable bladder injury; day 3 intravenous contrast-enhanced CT urography showed IPBR with leakage; day 5 laparoscopic repair/direct confirmation.	Laparoscopic bladder repair (multi-port)	Operative repair undertaken for IPBR; direct anatomical confirmation during repair.	5	No confirmed bladder-related complication	No repeat bladder repair	No in-hospital death
16	Day 0 trauma CT: bladder injury; same-day laparoscopic repair/direct confirmation.	Laparoscopic bladder repair (multi-port)	Operative repair undertaken for IPBR; direct anatomical confirmation during repair.	0	No confirmed bladder-related complication	No repeat bladder repair	No in-hospital death
17	Day 0 CT: hemoperitoneum; day 1 exploratory laparoscopy directly confirmed IPBR and repair was performed.	Laparoscopic bladder repair (single-port)	Operative repair undertaken for IPBR; direct anatomical confirmation during repair.	1	No confirmed bladder-related complication	No repeat bladder repair	No in-hospital death
20	Initial trauma CT/abdominopelvic CT followed by day 0 intravenous contrast-enhanced CT urography: EPBR with leakage; nonoperative catheter drainage.	Nonoperative catheter drainage	Nonoperative management; no bladder operation.	NA (nonoperative)	No confirmed bladder-related complication	No repeat bladder repair	No in-hospital death

Study ID	Diagnostic/confirmation pathway and timing	Treatment	Principal operative setting	Calendar days to initial operative bladder repair	Bladder-related complication	Repeat bladder repair	All-cause in-hospital mortality
24	Day 0 trauma CT: EPBR; nonoperative catheter drainage. Day 1 follow-up abdominopelvic CT showed continued extravasation during early nonoperative observation.	Nonoperative catheter drainage	Nonoperative management; no bladder operation.	NA (nonoperative)	No confirmed bladder-related complication	No repeat bladder repair	No in-hospital death
26	Day 0 trauma CT: EPBR; nonoperative catheter drainage.	Nonoperative catheter drainage	Nonoperative management; no bladder operation.	NA (nonoperative)	No confirmed bladder-related complication	No repeat bladder repair	No in-hospital death
27	Day 0 hematuria and CT hemoperitoneum; exploratory laparoscopy directly confirmed IPBR and same-day repair was performed.	Laparoscopic bladder repair (single-port)	Operative repair undertaken for IPBR; direct anatomical confirmation during repair.	0	No confirmed bladder-related complication	No repeat bladder repair	No in-hospital death
31	Day 0 trauma CT: EPBR; nonoperative catheter drainage.	Nonoperative catheter drainage	Nonoperative management; no bladder operation.	NA (nonoperative)	No confirmed bladder-related complication	No repeat bladder repair	No in-hospital death

Supplementary Table S5

De-identified patient-level characteristics, diagnostic and confirmation pathways, treatment, calendar-day repair interval, and in-hospital outcomes of all 46 unique patients.

Table S5 — Panel A. Injury characteristics.

Full cohort: IPBR, n = 21; EPBR, n = 25.

Study ID	Rupture site	Trauma type	Trauma mechanism	Pelvic fracture	Bladder AIS post-dot severity	Associated rectal/vaginal/bowel/mesenteric injury	Regional AIS profile
1	IPBR	Blunt	Fall from height	No	3	None documented in the re-abstracted categories	Chest AIS 3; Abdomen AIS 3; Pelvis/extremities AIS 2
2	IPBR	Blunt	Struck by blunt object	Yes	3	Colonic injury	Abdomen AIS 3; Pelvis/extremities AIS 3
3	EPBR	Blunt	Traffic accident	Yes	3	None documented in the re-abstracted categories	Abdomen AIS 3; Pelvis/extremities AIS 5; External AIS 1
6	EPBR	Blunt	Traffic accident	Yes	3	None documented in the re-abstracted categories	Head/neck AIS 2; Face AIS 2; Chest AIS 2; Abdomen AIS 3; Pelvis/extremities AIS 4; External AIS 1
7	EPBR	Blunt	Traffic accident	Yes	3	Rectal injury	Chest AIS 3; Abdomen AIS 2; Pelvis/extremities AIS 5
10	IPBR	Blunt	Struck by blunt object	No	3	None documented in the re-abstracted categories	Abdomen AIS 3
11	IPBR	Blunt	Fall from height	Yes	3	None documented in the re-abstracted categories	Chest AIS 2; Abdomen AIS 3; Pelvis/extremities AIS 2; External AIS 1
12	IPBR	Blunt	Traffic accident	No	3	None documented in the re-abstracted categories	Abdomen AIS 3
13	IPBR	Blunt	Ground-level fall	No	3	None documented in the re-abstracted categories	Abdomen AIS 3; External AIS 1
14	EPBR	Blunt	Traffic accident	Yes	3	None documented in the re-abstracted categories	Head/neck AIS 3; Face AIS 1; Chest AIS 5; Abdomen AIS 3; Pelvis/extremities AIS 2; External AIS 1
15	EPBR	Blunt	Fall from height	Yes	3	None documented in the re-abstracted categories	Chest AIS 3; Abdomen AIS 3; Pelvis/extremities AIS 5; External AIS 1
16	IPBR	Blunt	Traffic accident	Yes	3	None documented in the re-abstracted categories	Face AIS 1; Chest AIS 3; Abdomen AIS 3; Pelvis/extremities AIS 3; External AIS 1
17	IPBR	Blunt	Traffic accident	No	3	None documented in the re-abstracted categories	Head/neck AIS 3; Face AIS 2; Abdomen AIS 3; Pelvis/extremities AIS 2; External AIS 1
18	EPBR	Penetrating	Stab or incised injury	No	2	None documented in the re-abstracted categories	Abdomen AIS 2
20	EPBR	Blunt	Traffic accident	No	2	None documented in the re-abstracted categories	Chest AIS 2; Abdomen AIS 2
21	EPBR	Blunt	Traffic accident	Yes	3	None documented in the re-abstracted categories	Chest AIS 3; Abdomen AIS 3; Pelvis/extremities AIS 4; External AIS 1

Study ID	Rupture site	Trauma type	Trauma mechanism	Pelvic fracture	Bladder AIS post-dot severity	Associated rectal/vaginal/bowel/mesenteric injury	Regional AIS profile
22	EPBR	Penetrating	Stab or incised injury	No	2	Colonic and mesenteric injuries	Chest AIS 3; Abdomen AIS 3; External AIS 1
23	IPBR	Blunt	Traffic accident	No	3	Mesenteric injury	Chest AIS 3; Abdomen AIS 3; External AIS 1
24	EPBR	Blunt	Traffic accident	Yes	2	None documented in the re-abstracted categories	Chest AIS 3; Abdomen AIS 3; Pelvis/extremities AIS 4
25	EPBR	Blunt	Fall from height	Yes	3	None documented in the re-abstracted categories	Face AIS 2; Chest AIS 3; Abdomen AIS 3; Pelvis/extremities AIS 5; External AIS 1
26	EPBR	Blunt	Struck by blunt object	No	3	None documented in the re-abstracted categories	Abdomen AIS 3
27	IPBR	Blunt	Traffic accident	Yes	3	Colonic and mesenteric injuries	Chest AIS 3; Abdomen AIS 4; Pelvis/extremities AIS 3
28	IPBR	Blunt	Ground-level fall	No	3	None documented in the re-abstracted categories	Abdomen AIS 3
29	IPBR	Blunt	Traffic accident	No	3	None documented in the re-abstracted categories	Abdomen AIS 3
30	EPBR	Blunt	Traffic accident	Yes	4	Rectal, small-bowel, and mesenteric injuries	Abdomen AIS 4; Pelvis/extremities AIS 3
31	EPBR	Blunt	Fall from height	No	2	None documented in the re-abstracted categories	Abdomen AIS 2
34	IPBR	Blunt	Traffic accident	Yes	3	None documented in the re-abstracted categories	Abdomen AIS 3; Pelvis/extremities AIS 3; External AIS 1
35	EPBR	Blunt	Traffic accident	Yes	2	Vaginal injury	Abdomen AIS 2; Pelvis/extremities AIS 4
36	IPBR	Blunt	Traffic accident	No	3	None documented in the re-abstracted categories	Abdomen AIS 3; External AIS 1
37	IPBR	Blunt	Traffic accident	Yes	3	None documented in the re-abstracted categories	Head/neck AIS 5; Chest AIS 3; Abdomen AIS 3; Pelvis/extremities AIS 4
38	EPBR	Blunt	Fall from height	Yes	3	None documented in the re-abstracted categories	Chest AIS 3; Abdomen AIS 3; Pelvis/extremities AIS 4
39	EPBR	Blunt	Fall from height	Yes	2	None documented in the re-abstracted categories	Chest AIS 3; Abdomen AIS 3; Pelvis/extremities AIS 3
40	IPBR	Blunt	Traffic accident	No	3	None documented in the re-abstracted categories	Chest AIS 4; Abdomen AIS 3; Pelvis/extremities AIS 3; External AIS 1
41	EPBR	Blunt	Fall from height	No	3	None documented in the re-abstracted categories	Head/neck AIS 5; Face AIS 3; Chest AIS 3; Abdomen AIS 4; Pelvis/extremities AIS 5; External AIS 1
43	EPBR	Blunt	Fall from height	Yes	3	None documented in the re-abstracted categories	Head/neck AIS 4; Abdomen AIS 3; Pelvis/extremities AIS 2; External AIS 1
44	IPBR	Blunt	Unknown: found injured at home with facial bleeding and blood at the doorway; the patient could not recall the mechanism.	No	3	None documented in the re-abstracted categories	Abdomen AIS 3; Pelvis/extremities AIS 1; External AIS 1
45	EPBR	Blunt	Traffic accident	Yes	3	None documented in the re-abstracted categories	Abdomen AIS 3; Pelvis/extremities AIS 5
46	IPBR	Blunt	Traffic accident	No	3	None documented in the re-abstracted categories	Head/neck AIS 2; Face AIS 1; Abdomen AIS 3; Pelvis/extremities AIS 3; External AIS 1
49	IPBR	Blunt	Traffic accident	No	3	None documented in the re-abstracted categories	Abdomen AIS 3
50	IPBR	Blunt	Struck by blunt object	Yes	3	None documented in the re-abstracted categories	Chest AIS 2; Abdomen AIS 3; Pelvis/extremities AIS 5
51	EPBR	Blunt	Traffic accident	Yes	3	None documented in the re-abstracted categories	Abdomen AIS 3; Pelvis/extremities AIS 2; External AIS 2
52	EPBR	Blunt	Struck by blunt object	No	2	None documented in the re-abstracted categories	Abdomen AIS 2; Pelvis/extremities AIS 3; External AIS 1

Study ID	Rupture site	Trauma type	Trauma mechanism	Pelvic fracture	Bladder AIS post-dot severity	Associated rectal/vaginal/bowel/mesenteric injury	Regional AIS profile
54	IPBR	Blunt	Traffic accident	Yes	3	Mesenteric injury	Chest AIS 3; Abdomen AIS 3; Pelvis/extremities AIS 2
55	EPBR	Blunt	Traffic accident	Yes	3	None documented in the re-abstracted categories	Abdomen AIS 4; Pelvis/extremities AIS 4
56	EPBR	Blunt	Traffic accident	Yes	3	None documented in the re-abstracted categories	Abdomen AIS 3; Pelvis/extremities AIS 3
57	EPBR	Blunt	Traffic accident	Yes	3	Mesenteric injury	Head/neck AIS 2; Face AIS 1; Abdomen AIS 3; Pelvis/extremities AIS 4

Table S5 — Panel B. Diagnostic pathways, treatment, and in-hospital outcomes.

Full cohort: operative bladder repair, n = 42; nonoperative management, n = 4.

Study ID	Diagnostic/confirmation pathway and timing	Treatment	Principal operative setting	Calendar days to initial operative bladder repair	Bladder-related complication	Repeat bladder repair	All-cause in-hospital mortality
1	Day 0 trauma CT: IPBR; Day 2 laparoscopic repair/direct confirmation.	Laparoscopic bladder repair (single-port)	Operative repair undertaken for IPBR; direct anatomical confirmation during repair.	2	No confirmed bladder-related complication	No repeat bladder repair	No in-hospital death
2	Day 0 trauma CT: IPBR; same-day open repair/direct confirmation.	Open bladder repair	Operative repair undertaken for IPBR; direct anatomical confirmation during repair.	0	No confirmed bladder-related complication	No repeat bladder repair	No in-hospital death
3	Day 0 trauma CT: extraperitoneal hematoma; day 0 conventional cystogram demonstrated rupture; day 6 repair during pelvic fixation.	Open bladder repair	Pelvic/orthopedic surgery; bladder repair performed during pelvic fixation.	6	No confirmed bladder-related complication	No repeat bladder repair	No in-hospital death
6	Day 0 trauma CT followed by retrograde cystography through a Foley catheter in the angiography suite: EPBR; day 2 repair during pelvic surgery.	Open bladder repair	Pelvic/orthopedic surgery; bladder repair performed during pelvic surgery.	2	No confirmed bladder-related complication	No repeat bladder repair	No in-hospital death
7	Day 0 trauma CT recorded bladder rupture; PPP performed. Day 3 high drain output/elevated drain-fluid creatinine prompted re-exploration, direct identification of a 6-cm defect, and initial repair.	Open bladder repair	Re-exploration prompted by increased drain output and elevated drain-fluid creatinine; initial bladder repair performed.	3	Urinary leakage-related event: high drain output and elevated drain-fluid creatinine prompted re-exploration and initial bladder repair.	No repeat bladder repair	No in-hospital death
10	Day 0 contrast-enhanced trauma CT: IPBR; same-day open repair/direct confirmation.	Open bladder repair	Operative repair undertaken for IPBR; direct anatomical confirmation during repair.	0	No confirmed bladder-related complication	No repeat bladder repair	No in-hospital death
11	Day 0 trauma CT: suspected bladder rupture; day 3 laparoscopic repair/direct confirmation.	Laparoscopic bladder repair (multi-port)	Operative repair undertaken for IPBR; direct anatomical confirmation during repair.	3	No confirmed bladder-related complication	No repeat bladder repair	No in-hospital death
12	Day 0 trauma CT: IPBR; same-day exploratory laparotomy and repair/direct confirmation.	Open bladder repair	Operative repair undertaken for IPBR; direct anatomical confirmation during repair.	0	No confirmed bladder-related complication	No repeat bladder repair	No in-hospital death
13	Pre-admission outside abdominopelvic CT: hemoperitoneum and probable bladder injury; day 3 intravenous contrast-enhanced CT urography showed IPBR with leakage; day 5 laparoscopic repair/direct confirmation.	Laparoscopic bladder repair (multi-port)	Operative repair undertaken for IPBR; direct anatomical confirmation during repair.	5	No confirmed bladder-related complication	No repeat bladder repair	No in-hospital death
14	Day 0 trauma CT: pelvic-cavity hematoma without definite rupture; day 2 intravenous contrast-enhanced CT urography showed EPBR; same-day open repair/direct confirmation.	Open bladder repair	Bladder repair after preoperative intravenous contrast-enhanced CT urography documented EPBR.	2	Imaging-confirmed persistent urinary leakage; no repeat bladder repair.	No repeat bladder repair	All-cause in-hospital death 54 days after repair: acute respiratory distress syndrome; no direct bladder-related contribution documented.
15	Day 0 trauma CT: prevesical hematoma with hematuria; day 8 rupture directly identified and repaired during pelvic surgery.	Open bladder repair	Pelvic/orthopedic surgery; rupture identified during pelvic operation.	8	No confirmed bladder-related complication	No repeat bladder repair	No in-hospital death
16	Day 0 trauma CT: bladder injury; same-day laparoscopic repair/direct confirmation.	Laparoscopic bladder repair (multi-port)	Operative repair undertaken for IPBR; direct anatomical confirmation during repair.	0	No confirmed bladder-related complication	No repeat bladder repair	No in-hospital death
17	Day 0 CT: hemoperitoneum; day 1 exploratory laparoscopy directly confirmed IPBR and repair was performed.	Laparoscopic bladder repair (single-port)	Operative repair undertaken for IPBR; direct anatomical confirmation during repair.	1	No confirmed bladder-related complication	No repeat bladder repair	No in-hospital death
18	Day 0 trauma CT: bladder rupture; day 1 open repair/direct confirmation.	Open bladder repair	Bladder repair after preoperative imaging diagnosis.	1	No confirmed bladder-related complication	No repeat bladder repair	No in-hospital death

Study ID	Diagnostic/confirmation pathway and timing	Treatment	Principal operative setting	Calendar days to initial operative bladder repair	Bladder-related complication	Repeat bladder repair	All-cause in-hospital mortality
20	Initial trauma CT/abdominopelvic CT followed by day 0 intravenous contrast-enhanced CT urography: EPBR with leakage; nonoperative catheter drainage.	Nonoperative catheter drainage	Nonoperative management; no bladder operation.	NA (nonoperative)	No confirmed bladder-related complication	No repeat bladder repair	No in-hospital death
21	Day 0 CT: prevesical hematoma with hematuria, raising suspicion of bladder injury; day 3 rupture directly identified and repaired during pelvic surgery.	Open bladder repair	Pelvic/orthopedic surgery; rupture identified during pelvic operation.	3	No confirmed bladder-related complication	No repeat bladder repair	No in-hospital death
22	Day 0 shock after penetrating trauma; bladder rupture directly identified and repaired during emergency exploratory laparotomy.	Open bladder repair	Emergency exploratory laparotomy for penetrating polytrauma.	0	No confirmed bladder-related complication	No repeat bladder repair	No in-hospital death
23	Day 0 trauma CT: suspected IPBR; same-day exploratory laparotomy directly confirmed the rupture and repair was performed.	Open bladder repair	Operative repair undertaken for IPBR; direct anatomical confirmation during repair.	0	No confirmed bladder-related complication	No repeat bladder repair	No in-hospital death
24	Day 0 trauma CT: EPBR; nonoperative catheter drainage. Day 1 follow-up abdominopelvic CT showed continued extravasation during early nonoperative observation.	Nonoperative catheter drainage	Nonoperative management; no bladder operation.	NA (nonoperative)	No confirmed bladder-related complication	No repeat bladder repair	No in-hospital death
25	Day 2 PPP gauze removal: EPBR directly identified and repaired.	Open bladder repair	PPP gauze removal/change.	2	No confirmed bladder-related complication	No repeat bladder repair	No in-hospital death
26	Day 0 trauma CT: EPBR; nonoperative catheter drainage.	Nonoperative catheter drainage	Nonoperative management; no bladder operation.	NA (nonoperative)	No confirmed bladder-related complication	No repeat bladder repair	No in-hospital death
27	Day 0 hematuria and CT hemoperitoneum; exploratory laparoscopy directly confirmed IPBR and same-day repair was performed.	Laparoscopic bladder repair (single-port)	Operative repair undertaken for IPBR; direct anatomical confirmation during repair.	0	No confirmed bladder-related complication	No repeat bladder repair	No in-hospital death
28	Day 0 outside abdominopelvic CT: IPBR; same-day open repair/direct confirmation.	Open bladder repair	Operative repair undertaken for IPBR; direct anatomical confirmation during repair.	0	No confirmed bladder-related complication	No repeat bladder repair	No in-hospital death
29	Day 0 trauma CT: probable IPBR; same-day exploratory laparotomy and repair/direct confirmation.	Open bladder repair	Operative repair undertaken for IPBR; direct anatomical confirmation during repair.	0	No confirmed bladder-related complication	No repeat bladder repair	No in-hospital death
30	Day 0 emergency exploration directly identified extensive bladder/trigonal injury, but primary repair was not feasible because of injury severity; initial bladder repair was performed on day 6.	Open bladder repair	Perineal open-wound exploration for complex perineal injury.	6	Imaging-confirmed bladder-neck leakage with rectal fistulous communication.	Yes; day 14 after initial repair: inferior/perineal and anterior bladder-wall repair; concomitant soft-tissue debridement.	No in-hospital death
31	Day 0 trauma CT: EPBR; nonoperative catheter drainage.	Nonoperative catheter drainage	Nonoperative management; no bladder operation.	NA (nonoperative)	No confirmed bladder-related complication	No repeat bladder repair	No in-hospital death
34	Day 0 trauma CT: IPBR; same-day open repair/direct confirmation.	Open bladder repair	Operative repair undertaken for IPBR; direct anatomical confirmation during repair.	0	No confirmed bladder-related complication	No repeat bladder repair	No in-hospital death
35	Day 0 contrast-enhanced trauma CT: suspected bladder injury; day 5 rupture directly identified and repaired during pelvic surgery.	Open bladder repair	Pelvic/orthopedic surgery; rupture identified during pelvic operation.	5	No confirmed bladder-related complication	No repeat bladder repair	No in-hospital death
36	Day 0 trauma CT: possible IPBR; same-day open repair/direct confirmation.	Open bladder repair	Operative repair undertaken for IPBR; direct anatomical confirmation during repair.	0	No confirmed bladder-related complication	No repeat bladder repair	No in-hospital death
37	Day 0 contrast-enhanced trauma CT: IPBR; day 1 open repair/direct confirmation.	Open bladder repair	Operative repair undertaken for IPBR; direct anatomical confirmation during repair.	1	No confirmed bladder-related complication	No repeat bladder repair	No in-hospital death
38	Day 0 abdominopelvic CT: EPBR; day 10 rupture directly confirmed and repaired during internal iliac artery ligation.	Open bladder repair	Internal iliac artery ligation; concomitant bladder repair.	10	No confirmed bladder-related complication	No repeat bladder repair	No in-hospital death
39	Day 2 PPP gauze removal: EPBR directly identified and repaired.	Open bladder repair	PPP gauze removal/change.	2	No confirmed bladder-related complication	No repeat bladder repair	No in-hospital death
40	Day 0 trauma CT: IPBR; same-day open repair/direct confirmation.	Open bladder repair	Operative repair undertaken for IPBR; direct anatomical confirmation during repair.	0	No confirmed bladder-related complication	No repeat bladder repair	No in-hospital death
41	Day 0 hemodynamic instability prompted PPP; day 2 PPP gauze removal directly identified the bladder rupture and repair was performed.	Open bladder repair	PPP gauze removal/change.	2	No confirmed bladder-related complication	No repeat bladder repair	All-cause in-hospital death 2 days after repair: hypoxic brain injury/post-cardiac arrest syndrome with refractory hemodynamic instability; no direct bladder-related contribution documented.

Study ID	Diagnostic/confirmation pathway and timing	Treatment	Principal operative setting	Calendar days to initial operative bladder repair	Bladder-related complication	Repeat bladder repair	All-cause in-hospital mortality
43	Day 1 PPP gauze change/removal: EPBR directly identified and repaired.	Open bladder repair	PPP gauze removal/change.	1	No confirmed bladder-related complication	No repeat bladder repair	No in-hospital death
44	Day 0 abdominopelvic CT: IPBR; same-day open repair/direct confirmation.	Open bladder repair	Operative repair undertaken for IPBR; direct anatomical confirmation during repair.	0	No confirmed bladder-related complication	No repeat bladder repair	No in-hospital death
45	Day 1 preperitoneal pelvic packing: a 5-cm anterior-wall perforation was directly identified and repaired; the 200-mL bladder-filling leakage test was negative.	Open bladder repair	Bladder repair during preperitoneal pelvic packing for hemodynamic instability.	1	No confirmed bladder-related complication	No repeat bladder repair	No in-hospital death
46	Day 0 trauma CT: IPBR; day 1 open repair/direct confirmation.	Open bladder repair	Operative repair undertaken for IPBR; direct anatomical confirmation during repair.	1	No confirmed bladder-related complication	No repeat bladder repair	No in-hospital death
49	Day 1 trauma CT: suspected IPBR; cystoscopy confirmed rupture; same-day open repair.	Open bladder repair	Operative repair undertaken for IPBR; direct anatomical confirmation during repair.	1	No confirmed bladder-related complication	No repeat bladder repair	No in-hospital death
50	Day 0 trauma CT: IPBR; same-day open repair/direct confirmation.	Open bladder repair	Operative repair undertaken for IPBR; direct anatomical confirmation during repair.	0	No confirmed bladder-related complication	No repeat bladder repair	No in-hospital death
51	Day 1 perineal open-wound exploration: bladder rupture directly identified and repaired.	Open bladder repair	Perineal open-wound exploration.	1	No confirmed bladder-related complication	No repeat bladder repair	No in-hospital death
52	Day 0 emergency open inguinal/perineal wound exploration and bleeding control revealed an exposed bladder with an anterior-wall perforation; urologic repair was performed during the same operation.	Open bladder repair	Emergency open inguinal/perineal wound exploration and bleeding control; exposed bladder perforation repaired concomitantly.	0	No confirmed bladder-related complication	No repeat bladder repair	No in-hospital death
54	Day 0 trauma CT: suspected IPBR; day 1 cystography showed no extravasation; day 2 emergency surgery directly confirmed the rupture and initial repair was performed.	Open bladder repair	Operative repair undertaken for IPBR; direct anatomical confirmation during repair.	2	Residual urinary leakage directly observed during staged abdominal closure.	Yes; day 2 after initial repair: reinforcement for residual leakage during staged abdominal closure.	No in-hospital death
55	Day 0 abdominopelvic CT followed by day 1 intravenous contrast-enhanced CT urography: EPBR; day 7 repair with intraoperative bone spicule identification, followed by pelvic ORIF.	Open bladder repair	Pelvic/orthopedic surgery; bone spicule identified during bladder repair before pelvic ORIF.	7	Urinary leakage suspected from drain findings and elevated drain-fluid creatinine.	Yes; day 8 after initial repair: revision of bladder repair; concomitant pelvic fixation and wound debridement.	No in-hospital death
56	Day 16 pelvic/orthopedic approach: EPBR directly identified and repaired.	Open bladder repair	Pelvic/orthopedic surgery; rupture identified during the pelvic approach.	16	No confirmed bladder-related complication	No repeat bladder repair	No in-hospital death
57	Day 15 pelvic surgery: EPBR directly identified and repaired.	Open bladder repair	Pelvic/orthopedic surgery; rupture identified during pelvic surgery.	15	Drain findings prompted evaluation; cystography demonstrated posterior bladder leakage.	Yes; day 5 after initial repair: posterior bladder repair after cystography-confirmed leakage; concomitant pelvic wound revision/drain replacement/closure.	No in-hospital death
